# Alternatively spliced variants in Atlantic cod (*Gadus morhua*) support response to variable salinity environment

**DOI:** 10.1038/s41598-018-29723-w

**Published:** 2018-08-02

**Authors:** Agnieszka Kijewska, Magdalena Malachowicz, Roman Wenne

**Affiliations:** Department of Genetics and Marine Biotechnology, Institute of Oceanology Polish Academy of Sciences, Powstańców Warszawy 55, 81-712 Sopot, Poland

## Abstract

Analysis of gill transcriptome of the Atlantic cod from the Baltic Sea demonstrated that alternatively spliced (AS) variants may be actively involved in the process of adaptation to altered salinity. Some AS variants of different genes, like phospholipase A2 group IVC (PLA2G4C), appeared only in fish exposed to altered salinity, while other isoforms of the same genes were present in all experimental groups. Novel sequence arrangements represent 89% of all AS in the Baltic cod compared to the Atlantic population. Profiles of modified pathways suggest that regulation by AS can afford specific changes of genes expressed in response to the environment. The AS variants appear to be involved in the response to stress by modifications of signalling in apoptosis pathways, an innate immunological response and pro-inflammatory process. Present results support the hypothesis that developing new AS variants could support genome complexity and reinforce the ability to fast adapt to local environments.

## Introduction

Alternative splicing (AS) can drive determinative physiological change or can have a permissive role by providing mRNA variability that is used by other regulatory mechanisms^1^. AS is one of the most important cellular mechanisms in Eukaryota, generating multiple transcripts from a single gene, tissue-specific mRNA, modulating gene expression and function^2–4^. The variability in AS is so widespread that it can generate population-specific splicing ratios in human populations. Gonzàlez-Porta *et al*.^5^ found that up to 10% of the protein-coding studied AS variants exhibited different ratios in populations. Singh *et al*.^6^ found that in the cichlid fish, AS are related to ecological diversification. The splicing explains the discrepancy between a low number of genes and proteomic diversity^7–9^. Recent studies revealed that AS could affect physiological and developmental processes including organ morphogenesis^10^, the functioning of the immune system^11^ and neuronal development^12^.

Furthermore, adaptive transcriptional responses have been implicated in the evolution of tolerance to natural and anthropogenic stressors in the environment^13^. The altered expressions of spliced isoforms, linked to a stress response, were found in plants and animals^14–16^. Alternative splicing events have been found also in fish species like fugu (*Takifugu rubripes*), stickleback (*Gasterosteus aculeatus*), medaka (*Oryzias latipes*) and zebrafish (*Danio rerio*)^17^. AS were responsible for regulating developmental processes, anatomical structure formation, and immune system processes. Modifications of transcripts can also modulate the functionality of cellular components. Xu *et al*.^18^ postulated that some isoforms of membrane proteins can be deprived of transmembrane or membrane-associated domains and, as new soluble isoforms, can modulate the function of the membrane-bound forms.

Anatomical and physiological adaptations are based on genetic diversity and also post-transcriptional modifications^19,20^. Hashimoto *et al*.^21^ found that a hypertonic environment turned out to be an inducer of apoptosis in the epithelial cell line of a minnow (*Epithelioma Papulosum Cyprini*, EPC). This process also has a significant role in the extensive reorganization of mitochondria-rich cell populations during salinity acclimation accompanied by extensive remodelling of the gill epithelium^22,23^.

Although some mechanisms of response to salinity stress are well explored, very little is known about mechanisms that promote stress-induced variation leading to adaptations. This variation is interesting also because of interaction with metabolic pathways potentially involved in adaptation processes. Undoubtedly, AS variants may affect pathways involved in homeostasis in a cooperative or an antagonistic manner^24^. Also, there is a feedback mechanism of some pathways to regulate the alternative splicing machinery^25^.

The response to the suboptimal environmental conditions in the Baltic Sea is one of the key issues in studies of the Baltic cod (*Gadus morhua* L.). In the Baltic Sea, the Atlantic cod population structure is determined by salinity. The Baltic Sea is a young, semi-enclosed sea characterized by decreasing salinity in the northeastern direction (20 PSU to 6 PSU). Deepwater mass in the Baltic Sea has a higher salinity than surface waters. The average salinity in the Baltic Sea is about 8 PSU^26^. This low salinity limits the potential spawning areas for the Baltic cod to a few zones where the water has more than 14 PSU. The source of high salinity is the inflow of oceanic waters from the North Sea through the Danish Straits. Additionally, the central Baltic Sea is permanently stratified with a halocline located about 30–90 m below the surface.

During seasonal and diurnal migrations the Baltic cod is exposed to different salinities when crossing the halocline. Rapid changes in salinity during vertical migration of cod and during migration to spawning areas have been observed earlier^27^. In the process of adaptation to permanently lower salinity, the Baltic cod eggs have significantly higher buoyancy and spermatozoa mobility compared to fish from outside of the Baltic Sea^28^. Adaptation to specific environmental conditions (altered salinity levels) during seasonal and diurnal migrations is a vital and distinctive characteristic of the Baltic cod^19,29^.

The present analysis of the influence of AS variants on pathways, was conducted with the assumption that AS was to minimize stress in the Baltic cod during exposure to altered salinity and promote/stimulate adaptation to these suboptimal environmental conditions. A comprehensive analysis of AS in the Atlantic and the Baltic cod populations can be a step toward understanding the genome structure of this species. A set of Baltic cod reads from gill tissue, obtained using 454 pyrosequencing technology, was mapped to the Atlantic cod reference genome and all identified AS variants were analysed in the context of interaction with the suboptimal salinity, both lowered and elevated.

## Results

### Annotation and AS identification

A total of 962,516 reads with mean length 300–400 bp, representing 379 Mbp of Baltic cod gill tissue were obtained for transcriptome mapping and compared to the Atlantic cod reference transcripts. In effect, 61.32% of the Atlantic cod genes were recovered. All recovered genes were divided into biotypes. Most of them were classified as protein-coding genes (97.59%; 13,258), a low percentage of them (1.77%) were pseudogenes and 0.64% of sequences were non-coding. The number of reads and genes obtained for each experimental group was similar (Table 1).

**Table 1:** A summary of number of reads, bases and protein genes obtained for the Baltic cod transcriptome according to each experimental group.

Groups	CTRL		LS		RS		Total ± SD
	KIL	GDA	KIL	GDA	KIL	GDA	
Number of reads	159,733	158,860	160,002	162,249	158,613	163,060	160,419 ± 1,825
Bases (Mb)	63.1	63.4	63.6	63.6	63.1	62.7	63.25 ± 0.351
Genes	10,463	11,373	11,176	10,263	11,123	9,571	10,661 ± 689

CTRL – control group, LS – lowered salinity, RS – raised salinity. SD – standard deviation for differences between groups.

### AS variants analysis

In the data presented here, 3 933 AS events in 509 transcripts were documented (Supplementary Table S1). Among all transcripts, 55 had coverage above 100 (average 228.2; SD 175.5) and 454 were covered with less than 100 reads (average 27.2; SD 23.1).

**Figure 1:**
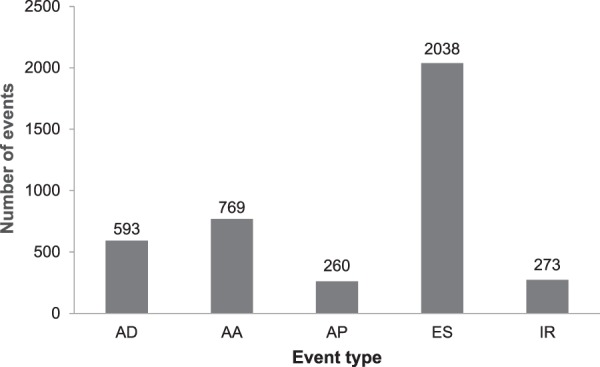
A number of AS events according to the events types.

Most of them (493 transcripts) had more than one event per gene (Table 2). Alternatively spliced events were also characterized by their type as defined in the ‘Materials’ section. Exon Skipping (ES) and Alternative Acceptor Site (AA) were the most frequent AS types (Fig. 1). Annotated transcripts, if doubled, had different sequences but their identity was based on the presence of domain and similarity to sequences from the Ensembl database. A total of 452 transcripts (89%) represented novel sequence arrangements compared to Atlantic cod and 11% were regarded as conserved.

**Table 2:** Statistics of AS variants and AS events in the Baltic cod transcriptome.

AS events per gene	No. of AS variants	No. of AS events	% of AS variants
1−9	369	1722	72.50
10−19	105	1385	20.63
≥ 20	35	826	6.88
Summary	509	3933	100

The Ensembl database searches and the BLAST analysis against non-redundant database identified 487 transcripts (95.67%) of AS transcripts had an annotation and 441 (86.64%) of them were described correctly. Only one isoform (transcription factor GATA-3) reported previously by Chi *et al*.^30^ for the Atlantic cod, was found in described transcripts from the Baltic cod.

**Figure 2:**
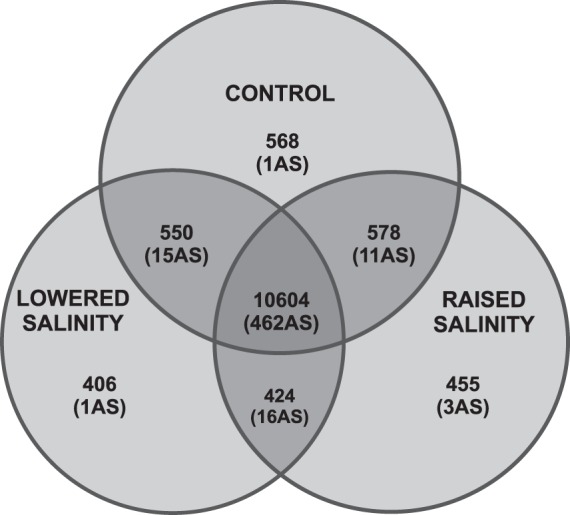
Venn diagram of shared transcripts and AS variants (in brackets) among Baltic cod experimental groups.

The comparison between the control (CTRL), lowered salinity (LS) and raised salinity (RS) groups of the Baltic cod, revealed some differences between the number of AS variants. The groups RS and LS shared 16 AS variants (3.14% of all AS variants). Furthermore, in LS, three original AS variants were observed, while in RS only one AS variant (Fig. 2). Both experimental groups (LS and RS) shared the part of AS variants with the control group (LS/CTRL = 11 variants, 2.16%, and RS/CTRL = 15 variants, 2.94%), where one splicing variant was present only in CTRL. From 47 AS variants found exclusively in one or two experimental groups, seven isoforms were already described in the Ensembl database. Some of the AS variants (15) were found exclusively in fish originating from two different samples from the Baltic Sea: Kiel Bight (KIL) and Gdańsk Bay (GDA) (Supplementary Table S2). In groups of RS and LS, three AS variants were found only in KIL, while one AS variant was identified in fish from GDA. Among AS variants identified in fish exposed to RS or LS two AS variants were found in the KIL sample and another one in the GDA sample. From the groups, RS/CTRL and LS/CTRL, four AS variants were found in the sample from KIL and another three AS variants were found exclusively in the GDA sample.

**Figure 3:**
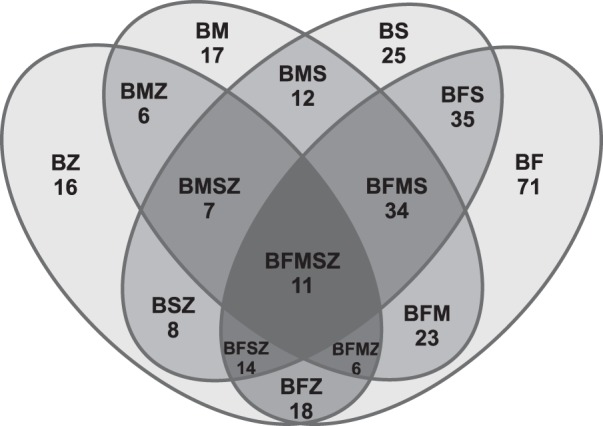
Venn diagram of shared AS variants among Baltic cod and four teleost species. For shared regions, B is Baltic cod, Z zebrafish, F fugu, M medaka and S stickleback. ‘BS’ represents the number of AS variants shared only between Baltic cod and stickleback.

A total of 303 AS transcripts in Baltic cod (39.41%) were shared with one or more of the four teleost species, and varied from 35.32% (107) in fugu, to 12.44% (37) in stickleback, and about 8.5% (25) in zebrafish and medaka. In addition, 174 AS variants (57.43%) were shared between two or more species. Between shared isoforms, 54 (9.57%) Baltic cod AS variants were conserved within euryhaline fish (medaka and stickleback; Fig. 3). The group of species with closer evolutionary relationships with each other: cod, fugu, medaka, stickleback, shared a larger number of AS gene identities with each other than with the more distantly related zebrafish (Fig. 3). An ontology definition was present for all 11 AS variants shared by all species. All of them represented ‘endomembrane system’ gene ontology (GO) category (p-value cut-off = 0.01)^31^. The frequencies of AS types were compared between the Baltic cod and the dataset presented by Lu *et al*.^17^. The Baltic cod genome was enriched in ES events (51.8% vs. 28.4–35.1%) and far fewer Intron Retention (IR) events (6.9%) than other species (16.8–25.9%).

### Gene ontology

**Figure 4:**
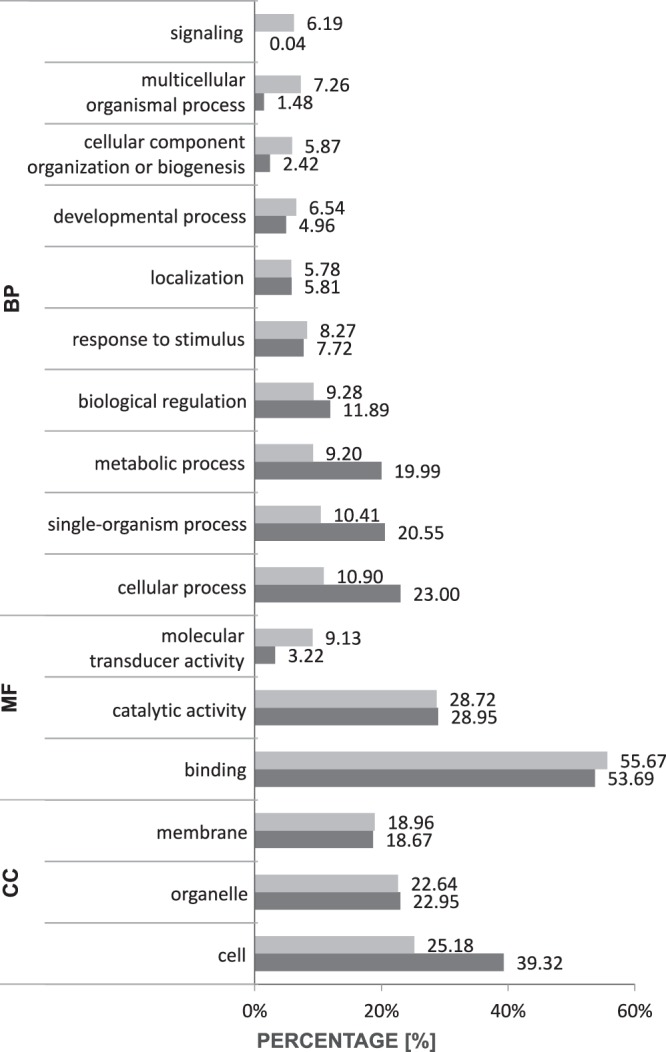
A percentage of annotated AS variants assigned to GO subcategories according to main GO categories. Light grey represents AS variants, while dark grey represents non-AS variants.

An ontology definition was present for 485 AS (98.38%) analysed with Blast2GO^31^. Isoforms were classified into three main GO categories (biological process – BP, molecular function – MF and cellular component – CC). Among those genes, 440 genes were assigned to at least one GO term in the BP category. The distribution of AS gene events in the category of BP did not differ greatly from non-AS transcripts (respectively 33.05% and 33.24%). In the CC category, non-AS transcripts constituted 18.42% of total share, while the percentage of AS transcripts was almost two times higher. The number of annotated AS transcripts in MF category was nearly 29% fewer compared to total annotated AS variants. Within the BP category, a ‘cellular process’ and a ‘single-organism process’ were the most dominant groups. In the CC category, a ‘cell’ was the dominant subcategory (24.75%), but its share was lower when compared to the share of all genes. Within the MF category, AS variants belonging to the ‘molecular transducer activity’ sub-category were more numerous than non-AS transcripts (9.13% vs. 3.22%) (Fig. 4).

In the gene-set analysis implemented in the ConsensusPathDataBase (CPDB)^32^, 99.7% of 393 genes were assigned to 18 molecular categories with q-value < 0.05, of which the most dominant were GO representing BP category (13 categories, Table 3). The most numerous GO sub-category was ‘protein binding’ representing 12.48% of transcripts. One of the smallest sub-categories, ‘transposase activity’ belonging to the MF category was represented by two of three gene transcripts which belong to this sub-category. GO categories were assigned separately for the dataset of transcripts found exclusively in experimental groups of Baltic cod. Annotations were found for 35 of 47 AS variants (gene of torsin family 1, tor1 was doubled). Classified transcripts were present in at least one GO sub-category: 20 AS variants in ‘single organism signalling’, and 23 AS variants in ‘cellular response to stimulus’ (both: BP level 2, with p = 0.01 and q < 0.05). The description was not available for AS variants with a high degree of sequence homology to natterin-like, caspase-like, amisyn-like and teleost multiple tissue opsin 3a. The description of their characteristics was based on the Zebrafish Information Network (ZFIN) electronic description and paper source^33^. The number of AS variants assigned to categories was correlated with response to a stimulus, and signalling, and related categories including metabolic processes and their regulation.

**Table 3:** Percentage of AS variants classified to sub-categories of gene ontology. P-value cut-off = 0.01.

Gene ontology term	candidates contained	p-value	q-value
GO:0006955 immune response	55 (3.5%)	0.0001	0.0066
GO:0050900 leukocyte migration	19 (5.4%)	0.0002	0.0066
GO:0045321 leukocyte activation	29 (4.2%)	0.0004	0.0105
GO:0009605 response to external stimulus	73 (3.0%)	0.0007	0.0143
GO:0051716 cellular response to stimulus	176 (2.5%)	0.0009	0.0143
GO:0065009 regulation of molecular function	79 (2.9%)	0.0012	0.0154
GO:0071704 organic substance metabolic process	248 (2.4%)	0.0015	0.0155
GO:0044238 primary metabolic process	241 (2.4%)	0.0016	0.0155
GO:0044237 cellular metabolic process	240 (2.4%)	0.0022	0.0187
GO:0042330 taxis	26 (3.7%)	0.0040	0.0315
GO:0044700 single organism signalling	153 (2.5%)	0.0049	0.0345
GO:0050789 regulation of biological process	250 (2.3%)	0.0054	0.0351
GO:0009056 catabolic process	64 (2.9%)	0.0065	0.0389
GO:0005789 endoplasmic reticulum membrane	36 (4.0%)	0.0002	0.0094
GO:0042175 nuclear outer membrane-endoplasmic reticulum membrane network	36 (3.9%)	0.0003	0.0094
GO:0016740 transferase activity	72 (3.3%)	0.0001	0.0032
GO:0005515 protein binding	254 (2.4%)	0.0004	0.0060
GO:0004803 transposase activity	2 (66.7%)	0.0013	0.0148

According to GO classification, among six identified AS genes from the eastern (GDA) group only, four were assigned to cation binding (MF level 3, q < 0.05), and metal ion binding (MF level 4, q < 0.05). Two of these genes were classified as ‘calcium ion binding’ (MF level 5, q < 0.05). In the western group (KIL), four genes represented hydrolase activity (MF level 2, q < 0.05) and three of them were assigned specifically to hydrolase activity, acting on ester bonds (MF level 3, q < 0.01). Two genes also represented nuclease activity (MF level 4, q < 0.01).

### Pathway analysis

AS variants shared between analysed species of fish: fugu, cod, zebrafish, medaka, and stickleback were mapped in Reactome database (five AS variants) and CPDB (seven AS variants). They were classified as: ‘haemostasis including platelet activation and degranulation’, ‘innate immune system’ with ‘toll-like receptor cascades’, and pathways involving arachidonic acid and its derivatives. AS variants mapped in Reactome were classified as belonging to ‘neutrophil degranulation’ pathway (FDR < 0.001; FDR – false discovery rate).

A total of 230 AS variants (52.27% of all annotated AS variants) were assigned to 12 pathways with q-value < 0.05 using CPDB (Table 4). Most of the pathways were doubled, depending on the model organism and database source, e.g. ‘bcr signalling’ in BioCarta database (www.biocarta.com), and ‘B Cell Receptor Signalling’ in Wikipathways database^34^. Pathways mainly represented: signalling and regulation processes, cell death processes, and inflammation processes.

**Table 4:** AS variants mapped in CPDB with overlap = 4, p < 0.01 (230 AS variants).

p-value	q-value	Pathway	Source	Members
0.000	0.035	RIPK1-mediated regulated necrosis	R	RIPK3; TNFSF10; FASLG; FADD
0.000	0.035	Regulated Necrosis	R	RIPK3; TNFSF10; FASLG; FADD
0.000	0.035	bcr signalling pathway	B	MAPK14; SYK; PLCG1; CAMK2B; BLNK
0.001	0.035	JAK-STAT pathway and regulation	I	TNFSF10; RYK; PRKAB1; PSMD5; IL12RB2; MAP3K7; IL16; MAPK14; SYK; RIPK3; AKT3; FASLG; CAMK2B; PSMC4; IL1R2; IL17RA
0.001	0.035	IL1	N	UBE2N; MAP3K7; MAPK14; DOK1; REL; IL1R2
0.001	0.035	B Cell Receptor Signalling Pathway	W	SYK; PIP5K1A; MAP3K7; MAPK14; PLCG1; REL; MEF2D; BLNK
0.001	0.035	mTORC1-mediated signalling	R	RRAGD; EIF4G1; AKT1S1; LAMTOR3
0.001	0.035	mTOR signalling	R	RRAGD; EIF4G1; AKT1S1; PRKAB1; LAMTOR3
0.001	0.035	Toll-like receptor signalling pathway	W	AKT3; CD40; MAP3K7; MAPK14; TLR1; FADD; TLR8; TLR9
0.001	0.035	PKB-mediated events	R	RRAGD; EIF4G1; AKT1S1; PRKAB1; LAMTOR3
0.002	0.037	Toll-like receptor signalling pathway - Homo sapiens (human)	K	TLR1; MAP3K7; CD40; MAPK14; AKT3; FADD; TLR8; TLR9
0.002	0.044	TNF signalling pathway - Homo sapiens (human)	K	FADD; MAP3K7; CSF1; MAPK14; RIPK3; AKT3; MMP9; EDN1

Pathways sources are indicated by letters: R- Reactome and K – KEGG, B – BioCarta, W – Wikipathways, I – INOH, N - NetPath.

**Figure 5:**
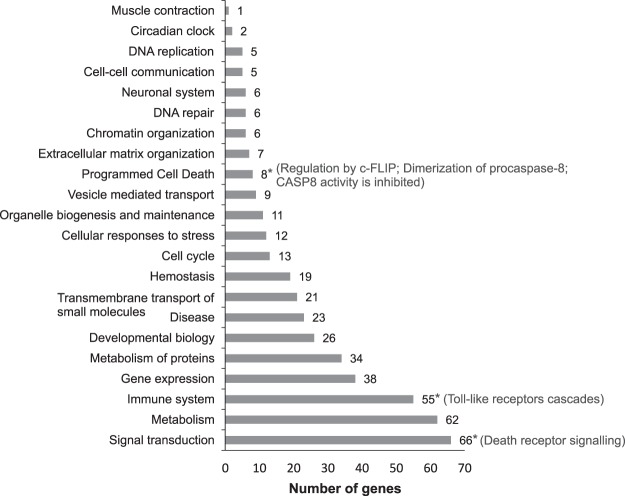
Molecular pathways classification of annotated 230 AS variants in the Reactome database. Asterisks mark the presence statistically significant (FDR < 0.05) sub-pathways (name of sub-pathways).

In turn, in the Reactome database^35^, most of 230 transcripts were mapped to the pathways: “signal transduction’, ‘metabolism’, ‘immune system’, and ‘gene expression’ (Fig. 5). About 27.5% of all AS variants and 46% of AS variants related to the metabolism were engaged in lipid metabolism. One AS variant of phospholipase A2 group IVC (PLA2G4C) was observed in all fish from the Baltic Sea. While another transcript of this enzyme was found only in Baltic cod exposed to shifted salinities (isoform indicated only in RS/LS group) (Supplementary Table S2).

The statistically significant pathways were the ‘RIPK1-mediated regulated necrosis’ (receptor interacting protein kinase 1- mediated regulated necrosis), ‘regulated necrosis’ and ‘TNF signalling’ (‘tumour necrosis factor signalling’) representing programmed cell death pathways. In the gills, the variants involved in these pathways was a new AS variant of RIPK3 (receptor interacting serine/threonine kinase 3 with complete domain) with complete domain but simultaneously with an AS variant of AKT3 (AKT serine/threonine kinase 3 with complete domain) (Supplementary Table S2). The AKT3 was also a part of ‘toll-like receptor signalling’ belonging to the ‘innate immune system’ category. There were also AS classified as representing ‘mTOR signalling’ and ‘JAK-STAT signalling’ pathways. This last pathway was represented by the most numerous group of genes, including transcripts of interleukin IL16 (interleukin 16) and interleukin receptors like IL1R2 (interleukin 1 receptor type 2), and IL12RB2 (interleukin 12 receptor subunit beta 2) (all with no domain) and IL17RA (interleukin 17 receptor A with complete domain).

From the experimental groups (RS, LS) seven AS variants were mapped with q < 0.05. A group of splicing variants shared by altered salinity (RS/LS) was represented by three AS variants. For example, eukaryotic translation initiation factor 4 gamma, 1 (EIF4G1 with complete domain) appeared in shifted salinities only (Supplementary Table S2).

Only eight AS variants present in the experimental groups and assigned to pathways were mapped with significant statistical support. Results obtained in CPDB mapped these AS variants to four main categories with q-value < 0.05. Four pathways represented the ‘immune system’ category and eight ‘signal transduction’ (Fig. 5).

Part of the mapped genes contained incomplete domains or not all domains. For example, in the present study; an AS variant of melastatin TRP channel (TRPM4, transient receptor potential cation channel subfamily M member 4), was found without a transmembrane-bound domain. Absence or presence of active domains is marked in Supplementary Table S2.

## Discussion

In fish genomes, about 16–43% of genes are alternative gene forms^17^ and probably constitute an important part of the functional proteome. AS of precursor mRNA (pre-mRNA) is an important gene regulation process that potentially regulates many physiological processes in eukaryotic cells, including the response to abiotic stresses such as salt stress^15^. The novelty of 89% AS variants suggests they could be characteristic of a Baltic population of cod. On the other hand, part of the AS variants could be expressed specifically in gills. So far, available data are insufficient to resolve this problem.

A comparison between the Baltic cod and other species analysed by Lu *et al*.^17^ indicated a group of universal AS present in all fish. All of them represented neutrophil degranulation pathway, a mechanism of the innate immune system consisting of the control of acute inflammation and pathogen killing^36^. Gills require very efficient mechanisms of defence, because of direct contact with environment and pathogens.

Besides shared AS variants, the data presented here revealed a low rate of AS events in gills (3.84%), which is about 4.5 and 3.7 times less than in the zebrafish and Atlantic cod genome, respectively^17^.

The use of gill tissue may have affected differences between this study and reference databases. It is highly probable that direct interactions between gills and environment affect the profile of transcriptome and can be different than in organs not directly exposed to the environment. Similar differences were observed previously in human transcriptome, where the majority of AS events were strongly correlated across tissues^37^. Support for this explanation is the lower number of protein coding AS variants observed in the gill transcriptome. The identified gill transcripts covered only 58% of the Atlantic cod transcriptome^38^. The expression of AS variants is restricted to limited tissue types present in gills (eg. epithelium)^39^.

It has been reported for humans that protein-coding AS variants exhibit low splicing variability within populations, with many AS variants exhibiting constant ratios across individuals^5^. The limited genetic variability reported for Baltic cod^40^ and loss of diversity caused by the selective pressure of adaptation to salinity could be also the reason for the low number of observed AS variants. Perhaps a positive effect on the suitability of specific AS variants was a part of the accelerated adaptation of the Baltic cod to a specific environment. In this context, the emergence, maintenance, and anchoring of specific AS variants should be considered as key points in pathways which affect their function and/or efficiency. This hypothesis is also supported by the presence of geographically original AS variants, obtained only from a single Baltic sample. The differences between observed isoforms and number of AS variants in the two Baltic groups of cod (KIL and GDA) may have been induced by ecological diversity^6^. A significantly lower amount of water-soluble cations probably enhances modifications of transcripts related to ionoregulation in eastern Baltic cod (GDA). In turn, irregular and rapid inflows of oceanic water into the west Baltic Sea^26^ (KIL group) favour the activity of hydrolases, probably involved in processes reducing stress like renewing of lipid damage in membranes, and DNA damage^13^ caused by osmotic stress. The ‘allopatric’ origin of these transcripts can be explained by differences between environmental profiling of the Baltic cod subpopulations as well as paralleled evolution of different transcripts in miscellaneous environmental conditions. This assumption is more probable due to the previous observation of Berg *et al*.^20^ who concluded that discrete parts of the Atlantic cod genome are subjected to directional selection and they are associated with adaptation to local environmental conditions. The Baltic Sea, with very differing local salinity conditions, was settled by the Atlantic cod probably because of the plasticity of cod’s genome, which is observed on many levels of genetic differentiation.

The dominance of some types of AS like ES could be an effect of the different arrangement of the Atlantic cod genome compared to other fish species^17^. It has been observed in teleost^17^ and other vertebrates^41^ that ES appears to be the most common AS type. The prevalence of this type of event is related to the length of upstream introns. According to Fox-Walsh *et al*.^42^, *Drosophila* and human exons with an upstream intron >4 kb were several-fold more susceptible to ES than exons with shorter upstream introns. This implies that in the Baltic cod, AS event types are, at least, partially determined by the characteristics of this species genome.

Mapped AS variants represented 22 pathways involved in ‘programmed cell death’, ‘immune system’ and ‘signal transduction’. It was expected that in cod crossing the halocline, hypo- or hypersalinity induces stress and simple cell damage caused by osmosis.

In Baltic cod, possible modifications of signalling pathways seem to be based more on the expression of AS variants of downstream genes than blocking or enhancing of the stimulus. In the Baltic cod, transcripts engaged with pathways related to death and survival of cells were revealed (Table 4) including TNF transcripts, and RIPK3, and AKT3. The ‘regulated necrosis’ pathway is observed in cells expressing RIPK3. The pathway is induced, among others, by TNF^43^. Cell activation of ‘TNF signalling’ pathways in response to tissue injury was observed in rainbow trout (*Oncorhynchus mykiss*)^44^. In turn, among AS variants associated with ‘TNF signalling’, AKT3 is considered one of the survival factors in cell death processes^45^. This composition of all AS variants suggests that cells could moderate their resistance to stress-induced death processes via the activation of the ‘AKT survival pathway’ as in the case of oxidative stress studied on Syk (spleen associated tyrosine kinase) deficient DT40 cells (chicken B-cell line)^46^.

One of the trigger signals activating defence mechanisms is the influence of abiotic stimuli on the integrity of membranes^13^. Lipid damage in membranes and DNA damage are also important macromolecular targets to the sensed stress^13^. The periodic but regular exposition of the Baltic cod to salinity shifts requires the intense activity of enzymes engaged in the lipid metabolism pathway. This hypothesis is supported by the presence of calcium-independent phospholipase A2γ encoded by PLA2G4C which plays a role in rebuilding damaged membranes in Atlantic salmon^47^. Presumably, efficient repairs of membrane damages limit the time and range of reaction to osmotic stress to local tissues or, in combination with modifications of signalling pathways, effectively limit the level of stress. Additionally, fatty acids are involved in the synthesis of eicosanoids^48^ which, like prostaglandins, affect osmotic water permeability in trout (*Salmo trutta*) and frogs (*Rana temporaria*)^49^.

Activation of cell defence mechanisms is the effect of transduction of signals both into the cell and between cells. Mapping of Baltic cod AS variants indicated that they are involved in the ‘JAK-STAT’ signalling pathway considered the principal signalling mechanism and activator of transcription^50,51^. Observed modifications of ‘JAK-STAT’ pathway mainly concern receptors of interleukins, which act as pro-inflammatory agents. The presence of AS variants with incomplete and complete domains related to the JAK-STAT and ‘mTOR’ pathways, suggest the AS variants are involved in regulation of catabolic and anabolic processes, including lipid metabolism and protein synthesis as observed by Manning and Cantley^52^.

Part of the AS variants associated with JAK-STAT were engaged also in the ‘B cell receptor signalling’ pathway. Stress directly affects the fish immune system due to immunosuppression and increased susceptibility to disease^53^. In the lowered salinity of the Baltic Sea, AS events found in the Baltic cod may modify B cell receptor signalling to endure the negative effects of lowered salinity (pro-inflammatory effects and weakened defence against pathogens in gills).

AS variants divided according to the experimental groups CTRL, LS and RS enriched already identified pathways and increased their statistical significance (Supplementary Table S2). Some AS variants (16) were observed in groups exposed to shifted salinity only (Fig. 2 and Supplementary Table S2). The presence of new AS variants in different experimental groups in alternative combinations (Supplementary Table S2) clearly indicates the active participation of AS variants in the regulation of response to imbalanced salinity. The presence of alternatively arranged isoforms, suggests that responses preventing the effects of salinity stress include the fast activation of AS variants which can play the role of auxiliary and regulatory units depending on their role in the specified path. For example, the presence of the transcript of EIF4G1 increased statistical support of ‘Insulin receptor signalling cascade’ to a significant level. This pathway activates signalling pathways leading to, among others, protein synthesis and nucleic acid (RNA and DNA) synthesis in fish^54^. The presence of an additional AS variant of this gene could enhance the appropriate reaction of the cells by increasing the synthesis of proteins supporting protection and survival.

Generally, the pathways and AS variants noted above suggest their influence on fast communication between cells, and cell survival mechanisms. AS variants could be a part of the mechanism of adaptation of the Baltic cod to a permanently lowered and periodically altered salinity. Adaptations have both genomic and post-transcriptional backgrounds, which increase the transcriptome complexity and plasticity under the pressure of environmental conditions. Although Berg *et al*.^20^ suggested that adaptation to low salinity promotes genomic divergence in Atlantic cod, the Baltic cod is genetically very little differentiated^40^. Probably, in the Baltic cod, unlike the western populations, AS variants could support genome complexity in the process of adaptation to the local environment.

Although AS could affect physiological and developmental processes of eukaryotic organisms^10–12^, it is necessary to answer questions about the role of AS variants in organismal adaptability to suboptimal conditions of the environment. Regulation of the pathways mentioned above by AS can result in rapid changes in expression of genes in response to the environment. Furthermore, AS can function as an ‘on/off’ switch by introducing premature termination codons, thereby directing mRNA degradation^19^. It is necessary to check the expression of detected AS variants. Further analysis of cod alternatively spliced variants is also necessary to test the functioning of pathways.

## Methods

### Fish and experiment protocol

Atlantic cod were collected by fyke net and pelagic trawl in November 2012 (n = 36) from KIL and GDA. Fish were transported to the Marine Station of the University of Gdańsk in Hel, Poland and were settled in tanks (2000 L). Fish were kept at 10 °C in recirculated water, which simulated the natural salinities of the geographic source of the Atlantic cod [original salinity of 18 PSU (KIL) and 8 PSU (GDA) from the place of fishing]. During the primary acclimatization period (over 14 days), fish were maintained at natural photoperiod and acclimated to laboratory conditions until they started feeding and displayed typical behaviour. Fish were fed once a day with fresh herrings during acclimatization and experimental periods. The salinity was changed gradually, by 1 PSU per hour, to minimize the acute stress of fish. After the salinity was changed in LS to 8 PSU (KIL) and 3PSU (GDA), and in RS to 28 PSU (KIL), and 18PSU (GDA), fish were maintained in the altered concentration of salt for 72 hours. In CTRL group, salinity remained unchanged. More details concerning the experiment are included in the publication of Kijewska *et al*.^29^.

After this period, samples for RNA (gills) were collected using sterile instruments. All experiments complied with EC Directive 2010/63/EU for animal experiments and were approved by the Local Ethics Committee on Animal Experimentation at Gdansk Medical University (decision no. 60/2012).

### RNA preparation and sequencing

Gills were collected from six individuals from each experimental group (LS, CTRL, RS) from KIL and GDA, and immediately submerged in RNAlater®, according to the manufacturer’s instruction (Qiagen, Hilden, Germany). Gills were stored at −80 °C prior to analysis. Before the extraction, tissues were defrosted on ice. Total RNA from each individual was extracted and purified with DNase using the ISOLATE II RNA Mini Kit (Bioline, London, UK) and was then stored at −80 °C. The concentration of extracted RNA (average 480 ng/µl) was determined at 260 nm on a microplate using the Epoch Microplate Spectrophotometer (BioTek Instruments, Inc., Winooski, USA). The ratio 260/280 was used for determination of the quality of RNA and results within a range of 1.8–2.15 were accepted. Each sample was checked using Agilent Bioanalyzer (Agilent, Santa Clara, CA, USA). Samples from six individuals with RNA integrity number (RIN) above seven were pooled for each experimental group (LS, CTRL, RS) from KIL and GDA separately. Pooled RNA was used for cDNA synthesis using the SMART (Switching Mechanism At 5’ end of RNA Template) kit from BD Biosciences Clontech. The cDNA normalization and pyrosequencing were performed by CD Genomics (USA), using Roche GS-FLX sequencing system according to the manufacturer’s instructions.

Baltic cod raw reads were deposited in the NCBI Sequence Read Archive (SRA) repository under the accession number SRP052904.

### Transcriptome annotation and AS identification

To characterize transcriptional events in the Baltic cod, all reads (≥50 nucleotides) were mapped onto the *G. morhua* reference genome using CLC Genomics Workbench (ver. 7.5.1, CLC Bio, Aarhus, Denmark) with default parameters. As a reference genome for transcriptome profiling, the genome of *G. morhua* from the Atlantic Ocean^55^, deposited in the Ensembl database, was used. Alignments with only one sequence coverage were excluded from analysis.

Information on predicted intron/exons obtained from the Atlantic cod Ensembl database was used to identify candidates and particular types of splicing events, based on sequence comparison. Only transcripts which were annotated as AS in Ensembl database were considered. Events with identical coordinates of an alternatively processed intron(s)/exon(s) compared to Atlantic cod Ensembl database were regarded as conserved. Description of the AS was based on the methodology of Wang *et al*.^37^. In the case of exon versus exon comparisons, where they had the same 3′ end but different 5′- ends they were classified as an Alternative Donor Site (AD); alternatively they were classified as AA. When both 3′- and 5′-end differed this event was classified as an Alternative Position Site (AP). An event was classified as ES when the exon was completely replaced by an intron. In contrast, if the specific intron remained unspliced, this case was classified as IR.

The transcripts annotations were downloaded from Ensembl database (release 87; http://www.ensembl.org/Gadus_morhua). However, due to lack of annotations in some identified AS transcripts, an additional comparison was performed against the NCBI non-redundant (NR) protein database using the BLASTX tool implemented in Blast + (v.2.2.29)^56^, with an E-value cut-off 10e^−10^. For functional annotation, GO terms^57^ were assigned to the AS transcripts using Blast2GO software^31^. The level 2 GO terms were retrieved and classified into three categories: CC, BP, and MF. Distribution of AS gene variants among the GO categories was compared to non-AS variants^38^. In CPDB the set of AS variants was searched for among the GO set. For each of the predefined sets, a p-value was calculated according to the hypergeometric test based on the number of physical entities present in both the predefined set and user-specified list of genes^32^. The *p*-values were corrected for multiple testing using the FDR method and presented as *q*-values in the results.

In addition, based on the AS gene names identified for fugu (*Takifugu rubripes*), medaka (*Oryzias latipes*), stickleback (*Gasterosteus aculeatus*), and zebrafish (*Danio rerio*)^17^, the potential level of conservation of annotated AS transcripts between the Baltic cod and this four teleosts was conducted.

### Pathway analysis

Most of the mapped transcripts were described as orthologues from the human genome database and identified with the HGNC (HUGO Gene Nomenclature Committee) symbol.

Annotated transcripts were analysed in Reactome V57 database^35^ and verified according to FDR values < 0.05, where the AS gene set was projected onto the human genome. Mapping was repeated in CPDB for human data and verified using *q*-values (*p*-value cut-off = 0.01 and minimum overlap = 4) and in Kyoto Encyclopedia of Genes and Genomes (KEGG) release 83.1, a database for human pathways and reference pathways^58^. KEGG pathways were assigned using the single directional best-hit (SBH) method in the KEGG Automatic Annotation Server (KAAS)^59^.

AS variants assigned to pathways were checked for the presence of specific domains and their integrity in Pfam database^60^.

## Electronic supplementary material

Supplementary Dataset 1

Supplementary Dataset 2

## References

[1] Kalsotra A, Cooper TA (2011). Functional consequences of developmentally regulated alternative splicing. Nat. Rev. Genet..

[2] Graveley BR (2001). Alternative splicing: increasing diversity in the proteomic world. Trends Genet..

[3] Maniatis T, Tasic B (2002). Alternative pre-mRNA splicing and proteome expansion in metazoans. Nature.

[4] Ruangsri J, Salger SA, Caipang CMA, Kiron V, Fernandes JMO (2012). Differential expression and biological activity of two piscidin paralogues and a novel splice variant in Atlantic cod (Gadus morhua L.). Fish Shellfish Immunol..

[5] Gonzàlez-Porta M, Calvo M, Sammeth M, Guigo R (2012). Estimation of alternative splicing variability in human populations. Genome Res..

[6] Singh P, Börger C, More H, Sturmbauer CH (2017). The Role of Alternative Splicing and Differential Gene Expression in Cichlid Adaptive Radiation. Genome Biol. Evol..

[7] Kim E, Goren A, Ast G (2007). Alternative splicing: current perspectives. BioEssays.

[8] Kim E, Magen A, Ast G (2007). Different levels of alternative splicing among eukaryotes. Nucleic Acids Res..

[9] Roy B, Haupt LM, Griffiths LR (2013). Alternative splicing (AS) of genes as an approach for generating protein complexity. Curr. Genomics.

[10] Moroy T, Heyd F (2007). The impact of alternative splicing *in vivo*: mouse models show the way. RNA.

[11] Vandiedonck C (2011). Pervasive haplotypic variation in the spliceo-transcriptome of the human major histocompatibility complex. Genome Res..

[12] Roberts JM (2013). Splicing factor TRA2B is required for neural progenitor survival. J. Comp. Neurol..

[13] Roelofs D, Morgan J, Stürzenbaum S (2010). The significance of genome-wide transcriptional regulation in the evolution of stress tolerance. Evol. Ecol..

[14] Råbergh CM (2000). Tissue-specific expression of zebrafish (*Danio rerio*) heat shock factor 1 mRNAs in response to heat stress. J. Exp. Biol..

[15] Ding F (2014). Genome-wide analysis of alternative splicing of pre-mRNA under salt stress in Arabidopsis. BMC Genomics.

[16] Yan K (2012). Stress-induced alternative splicing provides a mechanism for the regulation of microRNA processing in Arabidopsis thaliana. Mol. Cell.

[17] Lu J, Peatman E, Wang W (2010). Alternative splicing in teleost fish genomes: same – species and cross-species analysis and comparisons. Mol. Genet. Genomics.

[18] Xu XZS, Moebius F, Gill DL, Montell C (2001). Regulation of melastatin, a TRP-related protein, through interaction with a cytoplasmic isoform. Proc. Natl. Acad. Sci. USA.

[19] Pleiss JA, Whitworth GB, Bergkessel M, Guthrie C (2007). Rapid, transcript-specific changes in splicing in response to environmental stress. Mol. Cell.

[20] Berg PR (2015). Adaptation to low salinity promotes genomic divergence in Atlantic cod (*Gadus morhua* L.). Genome Biol. Evol..

[21] Hashimoto H, Matsuo Y, Yokoyama Y, Toyohara H, Sakaguchi M (1998). Induction of apoptosis in fish cells by hypertonic stress. Fish Sci..

[22] Kammerer B, Kültz D (2010). Prolonged apoptosis in mitochondria-rich cells of tilapia (*Oreochromis mossambicus*) exposed to elevated salinity. Comp. Biochem. Physiol. B Biochem. Mol. Biol..

[23] Kültz D, Li J, Gardell A, Sacchi R (2013). Quantitative molecular phenotyping of gill remodeling in a cichlid fish responding to salinity stress. Mol. Cell Proteomics.

[24] Chang MX, Zhang J (2017). Alternative Pre-mRNA Splicing in Mammals and Teleost Fish: A Effective Strategy for the Regulation of Immune Responses Against Pathogen Infection. Int. J Mol. Sci..

[25] Lee FFY, Chuang HC, Chen NY, Nagarajan G, Chiou PP (2015). Toll-like receptor 9 alternatively spliced isoform negatively regulates TLR9 signaling in teleost fish. PLoS One..

[26] Tomkiewicz, J., Lehmann, K.M., Stæhr, K. J. & St John, M. Oceanographic influences on the distribution of Baltic cod, Gadus morhua, during spawning in the Bornholm Basin of the Baltic Sea. Fisheries Oceanography **7**, 48–62 (1998).

[27] Neuenfeldt S, Hinrichsen H-H, Nielsen A, Andersen KH (2007). Reconstructing migrations of individual cod (*Gadus morhua* L.) in the Baltic Sea by using electronic data storage tags. Fisheries Oceanogr..

[28] Nissling A, Westin L (1997). Salinity requirements for successful spawning of Baltic and Belt Sea cod and the potential for cod stock interaction in the Baltic Sea. Mar. Ecol. Prog. Ser..

[29] Kijewska A (2016). Adaptation to salinity in Atlantic cod from different regions of the Baltic Sea. J. Exp. Mar. Bio. Ecol..

[30] Chi H (2012). Molecular characterizations and functional assessments of GATA – 3 and its splice variant in Atlantic cod (Gadus morhua L.). Dev. Comp. Immunol..

[31] Conesa A (2005). Blast2GO: a universal tool for annotation, visualization and analysis in functional genomics research+. Bioinformatics.

[32] Kamburov A (2011). ConsensusPathDB: toward a more complete picture of cell biology. Nucleic Acids Res..

[33] Gaudet, P., Livstone, M. & Thomas, P. The Reference Genome Project. Annotation inferences using phylogenetic trees. Automated Data Submission. http://zfin.org/ZDB-PUB-110330-1 (2010).

[34] Slenter DN (2017). WikiPathways: a multifaceted pathway database bridging metabolomics to other omics research. Nucleic Acids Res..

[35] Fabregat A (2016). The Reactome pathway Knowledgebase. Nucleic Acids Res..

[36] Havixbeck JJ, Barreda DR (2015). Neutrophil development, migration, and function in teleost fish. Biology.

[37] Wang ET, Sandberg R, Luo S (2008). Alternative isoform regulation in human tissue transcriptomes. Nature.

[38] Malachowicz M, Kijewska A, Wenne R (2015). Transcriptome analysis of gill tissue of Atlantic cod Gadus morhua L. from the Baltic Sea. Mar. Genomics.

[39] Laurent P, Dunel S (1980). Morphology of gill epithelia in fish. Am. J. Physiol. Regul. Integr. Comp. Physiol..

[40] Poćwierz-Kotus A (2015). Genetic differentiation of brackish water populations of cod Gadus morhua in the southern Baltic, inferred from genotyping using SNP – arrays. Mar. Genomics.

[41] Sugnet, C. W., Kent, W. J., Ares, M. & Haussler, D. Transcriptome and genome conservation of alternative splicing events in humans and mice. *Pac. Symp. Biocomput*. **9**, 66–77 (2004). 10.1142/9789812704856_0007 14992493

[42] Fox-Walsh KL (2005). The architecture of pre-mRNAs affects mechanisms of splice-site pairing. Proc. Natl. Acad. Sci. USA.

[43] Moriwaki K, Bertin J, Gough PJ, Orlowski GM, Chan FK (2015). Differential roles of RIPK1 and RIPK3 in TNF – induced necroptosis and chemotherapeutic agent-induced cell death. Cell Death Dis..

[44] Zou J (2003). Functional characterisation of the recombinant tumor necrosis factors in rainbow trout, Oncorhynchus mykiss. Dev. Comp. Immunol..

[45] Koseoglu S, Lu Z, Kumar C, Kirschmeier P, Zou J (2007). AKT1, AKT2 and AKT3 – dependent cell survival is cell line – specific and knockdown of all three isoforms selectively induces apoptosis in 20 human tumor cell lines. Cancer Biol. Ther..

[46] Ding J (2000). Syk is required for the activation of Akt survival pathway in B Cells exposed to oxidative stress. J. Biol. Chem..

[47] Bell JG, Farndale BM, Dick JR, Sargent JR (1996). Modification of membrane fatty acid composition, eicosanoid production, and phospholipase a activity in Atlantic Salmon (*Salmo salar*) Gill and Kidney by Dietary Lipid. Lipids.

[48] Smith WL (1992). Eicosanoid biosynthesis and mechanisms of action. Am. J. Physiol..

[49] Natochin YV (1998). Prostaglandin – dependent osmotic water permeability of the frog and trout urinary bladder. Comp. Biochem. Physiol. A Mol. Integr. Physiol..

[50] Aaronson DS, Horvath CM (2002). A road map for those who don’t know JAK-STAT. Science.

[51] Rawlings JS, Rosler KM, Harrison DA (2004). The JAK/STAT signaling pathway. J. Cell. Sci..

[52] Manning BD, Cantley LC (2007). AKT/PKB signaling: navigating downstream. Cell.

[53] Parra D, Reyes-Lopez FE, Tort L (2015). Mucosal Immunity and B Cells in Teleosts: Effect of Vaccination and Stress. Front. Immunol..

[54] Wood AW, Duan C, Bern HA (2005). Insulin like growth factor signalling in fish. Int. Rev. Cytol..

[55] Star B (2011). The genome sequence of Atlantic cod reveals a unique immune system. Nature.

[56] Camacho C (2009). BLAST+: architecture and applications. BMC Bioinformatics.

[57] Ashburner M (2000). Gene ontology: tool for the unification of biology. The Gene Ontology Consortium. Nat. Genet..

[58] Kanehisa M (2006). From genomics to chemical genomics: new developments in KEGG. Nucleic Acid Res..

[59] Moriya Y, Itoh M, Okuda S, Yoshizawa AC, Kanehisa M (2007). KAAS: an automatic genome annotation and pathway reconstruction server. Nucleic Acids Res..

[60] Finn RD (2016). The Pfam protein families database: towards a more sustainable future. Nucleic Acids Res..

